# A cost analysis of hCG trigger alone versus dual trigger for achieving live birth following *in vitro* fertilization

**DOI:** 10.1530/RAF-24-0095

**Published:** 2025-02-17

**Authors:** Esther H Chung, Arian Khorshid, Brindha Bavan, Ruth B Lathi

**Affiliations:** Stanford Fertility and Reproductive Health, Sunnyvale, California, USA

**Keywords:** dual trigger, hCG, GnRHa, decision analysis, IVF

## Abstract

**Graphical abstract:**

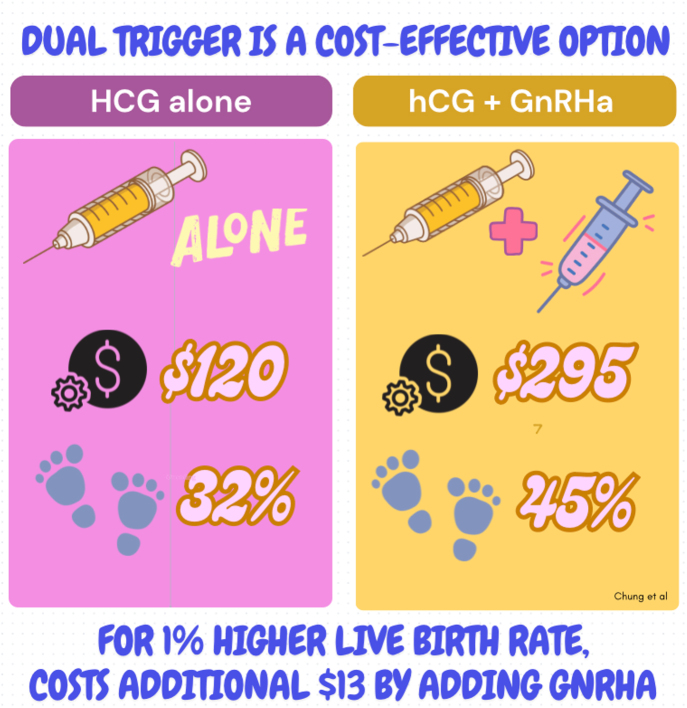

**Lay summary:**

In this study, we wanted to explore if adding gonadotropin agonist (GnRHa) to the standard use of human chorionic gonadotropin (hCG) for triggering egg maturation in *in vitro* fertilization (IVF) could be a more cost-effective option. What that means: Using hCG alone: Traditionally, hCG is the most common hormone used to trigger eggs to mature fully so that they can be collected for fertilization. Adding GnRHa: GnRHa is another medication that mimics a natural hormone produced by the brain. Adding this as a co-trigger may do better than just using hCG alone, by leading to more mature eggs and increasing live birth rates (LBRs). However, concerns about added costs and inconvenience remain. To address this, we created a cost model comparing LBRs and costs between the two strategies. Our analysis found that using this dual trigger increased LBRs by 13%, with a small cost increase of $175. For each 1% higher LBR, the added cost of using GnRHa + hCG was $13. Considering this minimal increase in cost relative to the overall high cost of IVF, using the dual trigger appears to be a cost-effective strategy to improve success rates for patients.

## Objective

Co-administration of human chorionic gonadotropin (hCG) and gonadotropin agonist (GnRHa) for triggering follicular maturation, as opposed to hCG alone, has been debated regarding its impact on *in vitro* fertilization (IVF) outcomes. GnRHa triggers the release of endogenous gonadotropins. Through a comprehensive literature review on PubMed using the following MeSH terms and keywords: ‘leuprolide’ OR ‘Lupron’ OR ‘GnRHa’, ‘dual trigger’ OR ‘co-trigger’, ‘hCG’, ‘IVF’ and ‘live birth’, we identified randomized controlled trials (RCTs) and meta-analyses that followed patients until the most pertinent outcome, live birth. These studies demonstrated that using a co-trigger with hCG (hCG + GnRHa) compared to hCG alone increases live birth rate (LBR) by 37% (relative risk (RR): 1.07–1.76) (Kim *et al.* 2014, Ali *et al.* 2020, Haas *et al.* 2020, Hu *et al.* 2021). Dual trigger was further associated with more mature oocytes retrieved without increasing ovarian hyperstimulation syndrome (OHSS), improved fertilization and more high-quality blastocysts.

Despite these benefits, concerns remain about the added cost and inconvenience of a dual trigger. There are no studies that confirm whether the comparative effectiveness justifies prescribing dual triggers in an already intensive process. Thus, we built a cost-based decision model to compare live birth probabilities and costs of the dual trigger (hCG + GnRHa) versus hCG trigger alone.

## Study design

**Figure 1:**
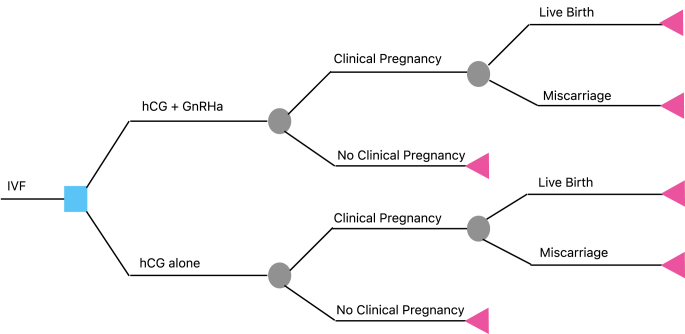
Cost-Analysis Decision Tree for the Two Trigger Strategies. Blue square is a decision node where a choice must be made between 2 alternatives: (i) dual trigger (hCG + GnRHa) and ii) hCG trigger alone; Gray circles are chance nodes representing probabilistic events with uncertain outcomes of no pregnancy, clinical pregnancy, miscarriage, or live birth; Pink triangles are terminal nodes indicating the final outcomes, including expected costs and consequences. Each branch represents a distinct decision pathway, incorporating probabilities and cost considerations. The tree structure provides a comparative analysis of potential scenarios.

A decision model was designed using TreeAge Pro Healthcare (2023R1.1; https://www.treeage.com) to compare live birth rates and costs between two strategies: i) dual trigger (hCG + GnRHa) and ii) hCG trigger alone (Fig. 1). The primary outcome was the cost per live birth. Probability inputs were based on studies that followed patients to live birth and controlled for appropriate confounders (Kim *et al.* 2014, Ali *et al.* 2020, Haas *et al.* 2020, Hu *et al.* 2021). Cost parameters were sourced from available pharmacy pricing (GoodRx 2022; ‘Prescription Prices, Coupons & Pharmacy Information’, n.d.), adjusted to 2022 $USD. Costs modeled from the healthcare perspective included all direct treatment costs but excluded intangibles such as time and transportation.

The model assumed normal responders, aged 18–41, with normal ovarian reserve (anti-Mullerian hormone >1 ng/mL and antral follicle count 6–20), and excluded patients at risk of OHSS. All patients were assumed to have completed one antagonist cycle with subsequent transfers, incurring comparable costs except for the difference in the trigger.

Probabilistic sensitivity analyses were conducted with 10,000 hypothetical patients, each undergoing 10,000 model iterations. Model parameters were varied using one-way sensitivity analyses based on plausible beta (LBR) and gamma (cost) distributions, defined by 95% CIs or calculated standard deviations.

## Results

The dual-trigger strategy incurred a $175 higher net cost ($295 vs $120, 95% CI: 173–178) compared to the hCG trigger strategy while resulting in a 13% higher live birth (45 vs 32%, 95% CI: 0.12–0.14) (Table 1). The dual trigger was costlier in 100% of model iterations with a higher LBR in 99.9% of iterations. Overall, the average incremental cost per 1% higher LBR was $13 when choosing the dual trigger compared to hCG alone. Sensitivity analyses demonstrated that the model was most sensitive to the cost of the GnRHa co-trigger.

**Table 1:** Comparing incremental cost per live birth for the two trigger strategies.

Strategy	Cost (USD)	Incremental cost	Effectiveness for live birth	Incremental effectiveness
hCG alone	120 (85–120)^5^		0.32 (0.28–0.31)^1–4^	
hCG + GnRHa	295 (260–1,035)^5^	175	0.45 (0.31–0.54)^1–4^	0.13

## Conclusion

This decision model shows that the dual trigger (hCG + GnRHa) improves the LBR per IVF cycle at a minimal cost increase. Assessing the costs of ‘add-ons’ in IVF is an important, responsible part of keeping overall costs down for patients. The cost of an IVF cycle, including medications, often exceeds $20,000. Adding GnRHa as a co-trigger increases that by less than 1%, and this increase appears justified. A limitation of this study is that the reliability of cost analyses is inherently limited by the quality and availability of prior RCTs used as clinical inputs. Based on existing data and our analysis, the dual trigger should be considered as a cost-effective option in IVF to help increase patients’ chances of live birth.

## Declaration of interest

E Chung is a medical advisor to LEVY Health. A Khorshid, B Bavan and R Lathi have no relevant disclosures.

## Funding

This work did not receive any specific grant from any funding agency in the public, commercial or not-for-profit sector.

## Author contribution statement

EC played a pivotal role in conceptualizing the research framework and methodology. She was primarily responsible for designing the study, developing the research question, performing the literature review and overseeing the process. In addition, she contributed significantly to the interpretation of the results and the write-up of the research letter. AK and BB contributed to refining the research design, performing the cost analysis and interpretation of the results and contributed to the write-up and editing of the manuscript. RL was critical in ideating the project and overseeing the process and, in addition, contributed to the write-up/editing of the research letter.

## Data availability

Data regarding any of the subjects in the study have not been previously published unless specified. Data will be made available to the editors of the journal for review or query upon request.
